# Altered Glymphatic‐Related MRI Metrics and Brain Network Reorganization in Drug‐Naive Children With Self‐Limited Epilepsy With Centrotemporal Spikes

**DOI:** 10.1002/cns.71079

**Published:** 2026-08-12

**Authors:** Yu Luo, Xiaoling Zhou, Jiajia Xu, Hua Lai, Qiang Lei, Yuwei Chen, Heng Liu

**Affiliations:** ^1^ Department of Radiology Affiliated Hospital of Zunyi Medical University Zunyi China; ^2^ Engineering Research Center of Intelligent Medical Imaging of Guizhou Provincial Department of Education Zunyi China; ^3^ Key Laboratory of Brain Function and Brain Disease Prevention and Treatment of Guizhou Province Affiliated Hospital of Zunyi Medical University Zunyi China; ^4^ Chengdu Women's and Children's Central Hospital, School of Medicine University of Electronic Science and Technology of China Chengdu China

**Keywords:** gBOLD‐CSF, glymphatic system, graph theory, mediation analysis, self‐limited epilepsy with centrotemporal spikes

## Abstract

**Objective:**

To explore cerebral waste clearance and its associations with brain network disturbances in drug‐naive children with self‐limited epilepsy with centrotemporal spikes (SeLECTS).

**Methods:**

We included 67 children with SeLECTS and 52 healthy controls (HC). All patients underwent standardized cognitive assessments. Metrics derived from T1WI, DTI, and fMRI were used to indirectly assess glymphatic‐related processes, including choroid plexus volume, perivascular space fraction, white matter free water (FW‐WM), and gBOLD‐CSF coupling. Functional brain networks were analyzed using graph theory. Linear models and partial correlation analyses were employed to examine between‐group differences in imaging metrics and their associations with cognitive function and topological properties. Additionally, the mediating effects of network properties were explored.

**Results:**

Compared with the HC group, the SeLECTS group exhibited increased FW‐WM and reduced gBOLD‐CSF coupling strength. Furthermore, gBOLD‐CSF coupling was negatively correlated with processing speed (*r* = −0.376, FDR*p* = 0.017). At the network level, the SeLECTS group exhibited a decreased clustering coefficient and characteristic path length, accompanied by increased global efficiency. The degree centrality and nodal efficiency values of the median cingulate and paracingulate gyri were significantly reduced and negatively correlated with gBOLD‐CSF coupling. In addition, nodal efficiency partially mediated the relationship between gBOLD‐CSF coupling and processing speed.

**Conclusion:**

These preliminary findings suggest that cognitive impairment in SeLECTS may be associated with the interplay between disturbances in cerebral waste clearance and brain network reorganization.

## Graphical abstract

Drug‐naive children with SeLECTS exhibit altered glymphatic‐related MRI metrics, including reduced gBOLD‐CSF coupling and increased FW‐WM, together with disrupted functional network topology. The gBOLD‐CSF coupling is associated with nodal properties in key regions and with cognitive performance. These findings suggest that impaired brain clearance and microenvironmental dysregulation may represent potential targets for future intervention.

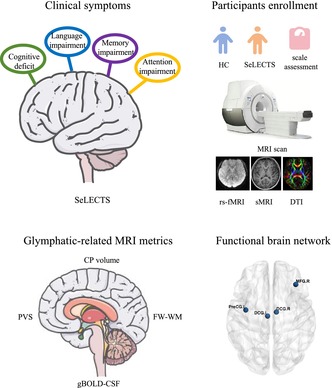

## 1 Introduction

Self‐limited epilepsy with centrotemporal spikes (SeLECTS) is the most common focal epilepsy syndrome in childhood [1]. Seizures typically occur during sleep and are characterized by high‐amplitude centrotemporal spikes on electroencephalography (EEG) [2]. Although SeLECTS is traditionally considered to have a favorable prognosis with age‐related remission, affected children may experience cognitive impairments involving language, memory, attention, and executive functions, which can adversely affect academic performance and social functioning [3]. Therefore, exploring the neuropathological mechanisms underlying these cognitive impairments is of significant clinical importance for improving prognosis.

Cerebrospinal fluid (CSF) circulation plays a critical role in clearing waste and maintaining brain homeostasis. In particular, the glymphatic system is a highly organized fluid transport system that facilitates the exchange of CSF and interstitial fluid (ISF), supporting the clearance of neurotoxic metabolites through perivascular spaces (PVS) and aquaporin‐4 (AQP4) channels localized on astrocytic endfeet. Glymphatic dysfunction has been linked to various neurological and neurodevelopmental disorders [4, 5]. In epilepsy, recurrent epileptiform activity induces neuroinflammatory responses and blood–brain barrier (BBB) disruption, which in turn may affect the neurovascular unit (NVU) and alter ISF homeostasis. These processes could disrupt AQP4 polarization through TGF‐β signaling pathways [6, 7, 8]. Animal studies have further demonstrated that glymphatic flow changes dynamically across different stages of epilepsy. During the acute phase, cytotoxic edema reduces the extracellular space volume and increases resistance to fluid movement, whereas chronic reactive gliosis contributes to the sustained suppression of CSF transport [9]. Furthermore, children with epilepsy exhibit increased visibility and dilation of Virchow‐Robin spaces, which may reflect alterations in perivascular fluid dynamics [10]. Collectively, these findings indicate a potential role for aberrant CSF dynamics and microenvironmental homeostatic disruption in the progression of epilepsy.

The direct assessment of glymphatic function in pediatric populations remains challenging, as conventional approaches are invasive and often poorly tolerated. Consequently, noninvasive MRI‐based methods have attracted increasing attention. Specifically, the choroid plexus (CP) is the primary source of CSF, forming the blood‐CSF barrier and mediating substance exchange and immune regulation [11]. White matter free water (FW‐WM) reflects the extracellular water content and is sensitive to multiple pathological processes, such as edema and inflammation [12]. PVS provide anatomical pathways for fluid movement and solute transport within the brain [13]. Additionally, gBOLD‐CSF coupling captures coordinated low‐frequency fluctuations between global brain activity and CSF flow [14]. These complementary imaging markers are considered to indirectly reflect glymphatic‐related processes. Although alterations in these imaging metrics have been reported in various types of epilepsy [15, 16], their utility in children with SeLECTS remains to be elucidated. Thus, a comprehensive multiparametric evaluation should be performed in this population.

Furthermore, SeLECTS is associated with widespread alterations in brain network organization rather than abnormalities confined to the epileptogenic focus, potentially contributing to the observed cognitive deficits. Graph theory analysis provides a powerful framework for characterizing such system‐level alterations by modeling the brain as a complex network composed of nodes and edges, enabling the evaluation of abnormal changes at the global and nodal levels [17]. Emerging evidence indicates that glymphatic dysfunction, which results in impaired waste clearance and toxic substance accumulation, may exacerbate abnormalities in brain networks [18, 19]. However, knowledge of their interplay in children with SeLECTS is limited.

Therefore, the present study aims to integrate multiple MRI‐derived metrics, including CP volume, PVS fraction, FW‐WM, and gBOLD‐CSF coupling, to evaluate CSF flow dynamics in drug‐naive children with SeLECTS. By investigating the potential associations among cerebral clearance, network topology, and cognitive performance, we hope to provide novel insights into the neural basis of cognitive dysfunction in children with SeLECTS.

## 2 Materials and Methods

### 2.1 Participants

Between January 2023 and December 2025, we included 67 children diagnosed with SeLECTS from our hospital. The inclusion criteria were as follows: (1) meeting the diagnostic criteria of the International League Against Epilepsy (ILAE), (2) no significant abnormalities on conventional MRI, (3) age between 6 and 14 years, and (4) no prior antiepileptic drug therapy. The exclusion criteria were as follows: (1) comorbid neurological or psychiatric disorders affecting cognition, (2) contraindications to MRI scanning, and (3) poor image quality affecting subsequent analyses. Additionally, 52 age‐, sex‐, and education‐matched healthy controls (HC) were included. All participants were right‐handed. Written informed consent was obtained from the legal guardians of all participants. Patients with SeLECTS were assessed by experienced pediatricians using the Wechsler Intelligence Scale for Children‐Fourth Edition (WISC‐IV). The assessment covered multiple cognitive domains, including verbal comprehension (VCI), perceptual reasoning (PRI), working memory (WMI), and processing speed (PSI). These indices were combined to derive the full‐scale intelligence quotient (FSIQ), which provides an estimate of general intellectual ability. Patients' clinical history was obtained from medical records. Disease duration was defined as the interval between initial seizure onset and the date of MRI acquisition. The total number of seizures was defined as the cumulative number of seizure episodes from disease onset to MRI scanning.

### 2.2 MRI Scan

We utilized a 3.0 T MRI scanner (Siemens Healthineers, Germany) for imaging data acquisition. The 3D‐T1WI sequence employed TR/TE = 1570/2.4 ms, slice thickness = 1.0 mm, number of slices = 146, voxel size = 1.0 × 1.0 × 1.0 mm^3^, and FOV = 256 mm × 256 mm. The DTI sequence used TR/TE = 9700/76 ms, FOV = 256 mm × 256 mm, slice thickness = 2.0 mm, slice gap = 0.6 mm, *b*‐values = 0 or 1000 s/mm^2^, and diffusion direction = 64. The rs‐fMRI sequence employed TR/TE = 2000/30 ms, flip angle = 80^°^, thickness/gap = 2.0/0.66 mm. Foam pads were used to minimize head motion, and participants were instructed to keep their eyes closed, stay awake, and remain relaxed during the scan.

### 2.3 CP Volume Calculation

**FIGURE 1:**
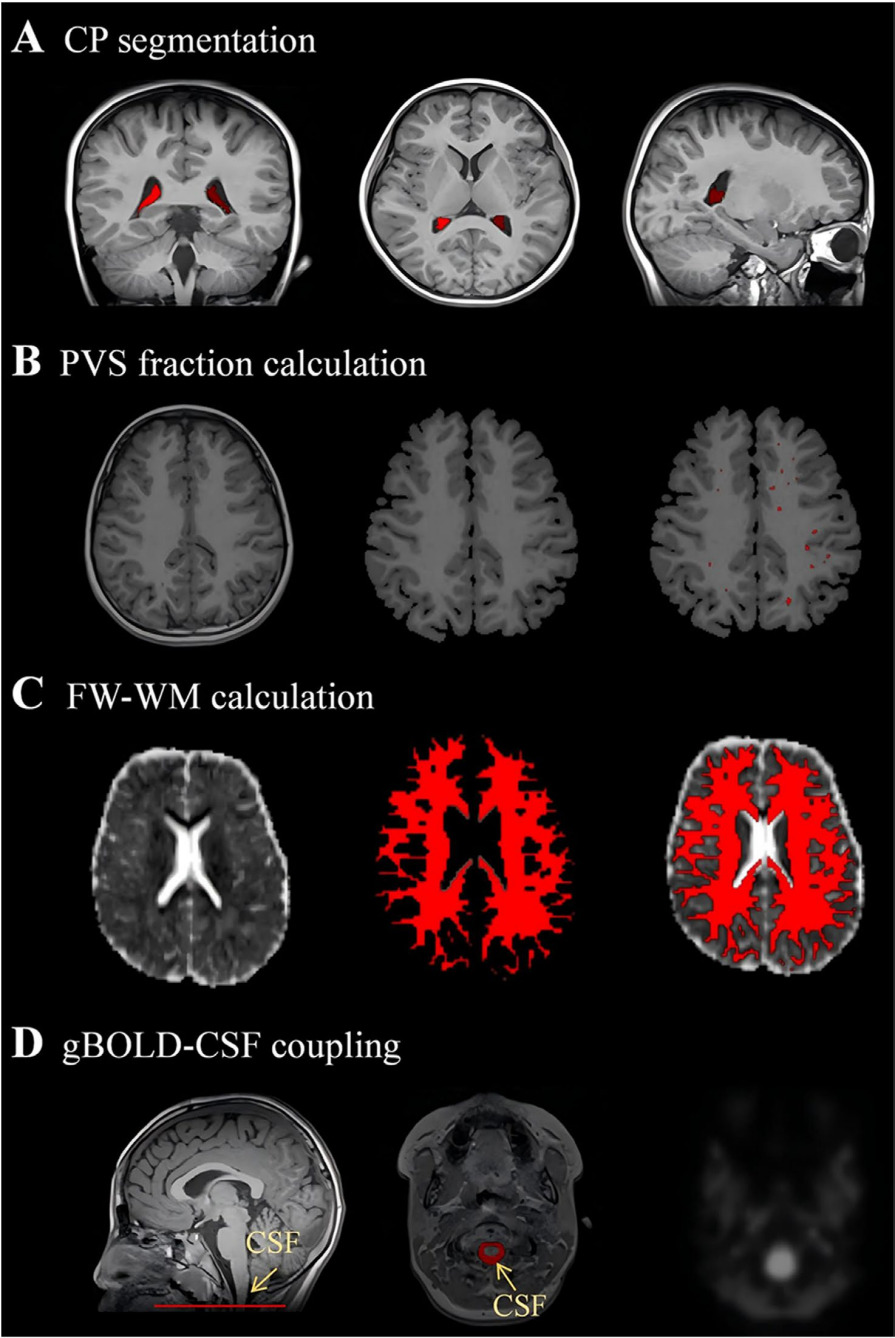
Schematic diagram of glymphatic‐related MRI metrics calculation. (A) T1WI was registered to standard space, with the choroid plexus segmented using a pre‐trained 3D U‐Net. (B) PVS fraction was derived using a Frangi filter and normalized to total intracranial volume. (C) FW maps were generated using bitensor diffusion modeling, with mean FW calculated within the white matter mask excluding PVS regions. (D) Cortical gray matter (Harvard‐Oxford atlas) and CSF ROIs (red) were defined, from which gBOLD and CSF signals were extracted. CP, choroid plexus; CSF, cerebrospinal fluid; FW, free water; PVS, perivascular spaces; ROI, region of interest.

Prior to further image preprocessing, all acquired MRI data underwent visual quality inspection to exclude significant artifacts and anomalies. We employed a previously validated deep learning approach based on a 3D U‐Net model for CP segmentation [20]. Briefly, T1WI data were preprocessed and registered to the MNI152 template. A probabilistic atlas of the CP was used to crop the registered images, thereby confining subsequent analyses to consistent regions of interest (ROIs) across all subjects. Automated segmentation within these regions was performed using the pre‐trained 3D U‐Net model, and the resulting masks were used to extract CP volume in standard space (Figure 1).

### 2.4 PVS Fraction Calculation

PVS mapping was performed on the T1WI data using an established approach [21]. First, WM masks were extracted from the T1WI data using FreeSurfer. Subsequently, a Frangi filter was applied to the preprocessed images using the Quantitative Imaging Toolkit, with parameter settings (α = 0.5, β = 0.5, C = half the maximum Hessian norm) consistent with those of previous studies [12]. A PVS probability map was generated by performing maximum likelihood estimation of vesselness across multiple scales. To mitigate the influence of outliers, standard normalization and binarization procedures were employed to derive a binary PVS mask. Finally, the PVS fraction was calculated as the ratio of PVS volume within the WM region to the total intracranial volume and then used for subsequent statistical analysis.

### 2.5 FW‐WM Calculation

For each participant, DTI data were preprocessed using the FMRIB Software Library (FSL). The preprocessed data were then analyzed using the Diffusion Imaging in Python (Dipy) algorithm to generate whole‐brain FW maps by implementing a bitensor model. The mean FW value was calculated within the WM mask for each participant, excluding the PVS regions.

### 2.6 gBOLD‐CSF Coupling Analysis

Rs‐fMRI data were preprocessed using the DPABI toolbox within the SPM12 framework. The raw images were converted to NIfTI format, followed by the removal of the first 10 volumes to allow for magnetization equilibrium and subsequent slice timing correction. For fMRI signal extraction, the data underwent head motion correction, spatial smoothing (FWHM = 4 mm), linear detrending, and temporal band‐pass filtering (0.01–0.1 Hz). Participants with head motion exceeding 3 mm of translation or 3° of rotation were excluded. Consistent with previous studies, nuisance regression of the global BOLD signal, CSF signal, and motion parameters was not performed to preserve critical hemodynamic information, which was the primary focus of the present analysis [22, 23, 24]. Cortical gray matter ROIs were defined based on the Harvard‐Oxford cortical structural atlas and inversely transformed into each subject's native fMRI space to minimize registration‐related signal loss [25]. The signals derived from each subject's gray matter mask were normalized to z‐scores and averaged for subsequent analyses. For CSF signal extraction, the rs‐fMRI data underwent linear detrending and temporal band‐pass filtering (0.01–0.1 Hz). The CSF signal was extracted from a manually defined ROI at the cerebellar bottom on functional images after verification of its anatomical location on the corresponding T1‐weighted structural images. ROI delineation was performed independently by two experienced radiologists blinded to clinical information, and inter‐observer reliability was assessed using the intraclass correlation coefficient (ICC).

To explore the dynamic coupling between gBOLD and CSF signals, we calculated their temporal cross‐correlation using Pearson's correlation across time lags ranging from −10 s to +10 s, with sensitivity analyses conducted using different windows. In our study, the negative peak observed at +4 s and the positive peak at −4 s exhibited similar amplitudes. Therefore, the maximum negative peak at +4 s was used for subsequent analyses. We further calculated the cross‐correlation function between the negative derivative of the gBOLD signal and the CSF signal [14]. A permutation‐based approach (10,000 iterations) was implemented to evaluate statistical significance, generating corresponding null distributions. All computations were performed using MATLAB (version R2022b).

### 2.7 TBSS Analysis

The DTI data were preprocessed using FSL (version 6.0). Preprocessing steps included denoising, the removal of Gibbs ringing artifacts, correction for head motion and eddy currents, and bias field correction. Skull stripping was performed to generate a brain mask. A bitensor model was subsequently applied to obtain the FW map and FW‐corrected diffusion tensor maps, including FW‐corrected fractional anisotropy (FAt) and FW‐corrected mean diffusivity (MDt). TBSS analysis was performed following the standard pipeline, including nonlinear registration and projection onto the mean FA skeleton using a threshold of 0.2. Voxel‐wise group comparisons between patients and controls were conducted using FSL randomize with 5000 permutations, with age, sex, and education included as covariates. Correction for multiple comparisons was performed using threshold‐free cluster enhancement (TFCE) with a family‐wise error (FWE) corrected threshold of *p* < 0.05.

### 2.8 Functional Brain Network Analysis

Rs‐fMRI data preprocessing and functional network construction were performed using the DPABI and GRETNA toolboxes: (1) data format conversion, (2) removal of the first 10 time volumes, (3) slice timing correction, (4) head motion correction, excluding participants with head motion exceeding 3 mm in translation or 3° in rotation, as well as those with a mean framewise displacement (FD) > 0.2 mm, (5) co‐registration of the realigned functional images to each subject's high‐resolution 3D T1 structural image, followed by spatial normalization to the MNI standard space and resampling to 3 × 3 × 3 mm^3^ voxels, (6) linear detrending, (7) nuisance regression of WM signals, CSF signals, and motion parameters, and (8) temporal band‐pass filtering (0.01–0.08 Hz) to reduce low‐frequency drift and high‐frequency physiological noise.

A total of 116 brain regions defined by the AAL atlas served as network nodes. The mean time series of each region was extracted to generate a 116 × 116 Pearson correlation matrix for each participant. Each matrix was binarized at multiple sparsity thresholds ranging from 0.05 to 0.40 (step size: 0.01) [26]. The area under the curve (AUC) for each network metric was then calculated. Global network properties included small‐worldness (*σ*), clustering coefficient (Cp), normalized clustering coefficient (γ), characteristic path length (Lp), normalized characteristic path length (λ), global efficiency (Eg), and local efficiency (Eloc). At the nodal level, we computed degree centrality (Dc) and nodal efficiency (NE). Group comparisons of network properties were conducted using two‐sample *t*‐tests, with age, sex, education, and mean FD included as covariates. To ensure the robustness of the observed group differences, these metrics were additionally calculated over alternative sparsity ranges (0.05–0.35 and 0.10–0.40 with a step size of 0.01) for validation. Statistical significance for global and nodal metrics was set at *p* < 0.05 and Bonferroni‐corrected *p* < 0.05, respectively. Brain regions with significant group differences were visualized using the BrainNet Viewer toolbox.

### 2.9 Statistical Analysis

Demographic analysis was performed using SPSS 26.0 software (IBM Corp., Armonk, NY, USA). Continuous data were expressed as mean ± standard deviation (SD) and compared between groups using the two‐sample *t*‐test (for normally distributed data) or the Mann–Whitney *U* test (for non‐normally distributed data). Categorical variables were analyzed using the chi‐square test. The reliability of gBOLD‐CSF coupling measurements within the CSF ROI was assessed using ICC analysis, and the mean value was used for subsequent analyses. Group differences in the four MRI‐derived metrics (CP volume, PVS fraction, FW‐WM, and gBOLD‐CSF coupling) were assessed using a general linear model, and partial correlations were used to examine the relationships between these imaging metrics and both clinical scales and network properties. Both analyses controlled for age, sex, and education, and multiple comparisons were corrected using the false discovery rate (FDR) method with a significance threshold of *p* < 0.05. Detailed results of all correlation analyses are provided in Tables S1 and S2. Mediation models were tested using the SPSS PROCESS macro (version 3.5) with 10,000 bootstrap resamples to derive 95% confidence intervals. A significant mediation effect was identified when the interval did not include zero.

## 3 Results

### 3.1 Clinical Data

Ultimately, we included 67 patients and 52 HC. There were no significant differences in age (*p* = 0.211), sex (*p* = 0.575), education (*p* = 0.221), and mean FD (*p* = 0.304) between the two groups (Table 1).

**TABLE 1:** Demographic characteristics of the HC and SeLECTS groups.

Characteristics	HC (*n* = 52)	SeLECTS (*n* = 67)	*p*
Male/female (*n*)	32/20	37/30	0.575
Age (years)	8.604 ± 1.756	8.196 ± 1.630	0.211
Education (years)	2.940 ± 1.720	2.610 ± 1.749	0.221
Mean FD	0.084 ± 0.026	0.081 ± 0.032	0.304
Disease duration (days)	—	70.190 ± 96.157	—
Total number of seizures	—	2.040 ± 1.522	—
VCI	—	93.580 ± 12.787	—
PRI	—	99.120 ± 9.315	—
WMI	—	93.490 ± 10.087	—
PSI	—	98.700 ± 7.065	—
FSIQ	—	94.720 ± 11.143	—

*Note:* Data are presented as mean ± SD. Abbreviations: FD, framewise displacement; FSIQ, full‐scale intelligence quotient; HC, healthy controls; PRI, perceptual reasoning index; PSI, processing speed index; SeLECTS, self‐limited epilepsy with centrotemporal spikes; VCI, verbal comprehension index; WMI, working memory index.

### 3.2 gBOLD Signal Couples With CSF Signal Changes

**FIGURE 2:**
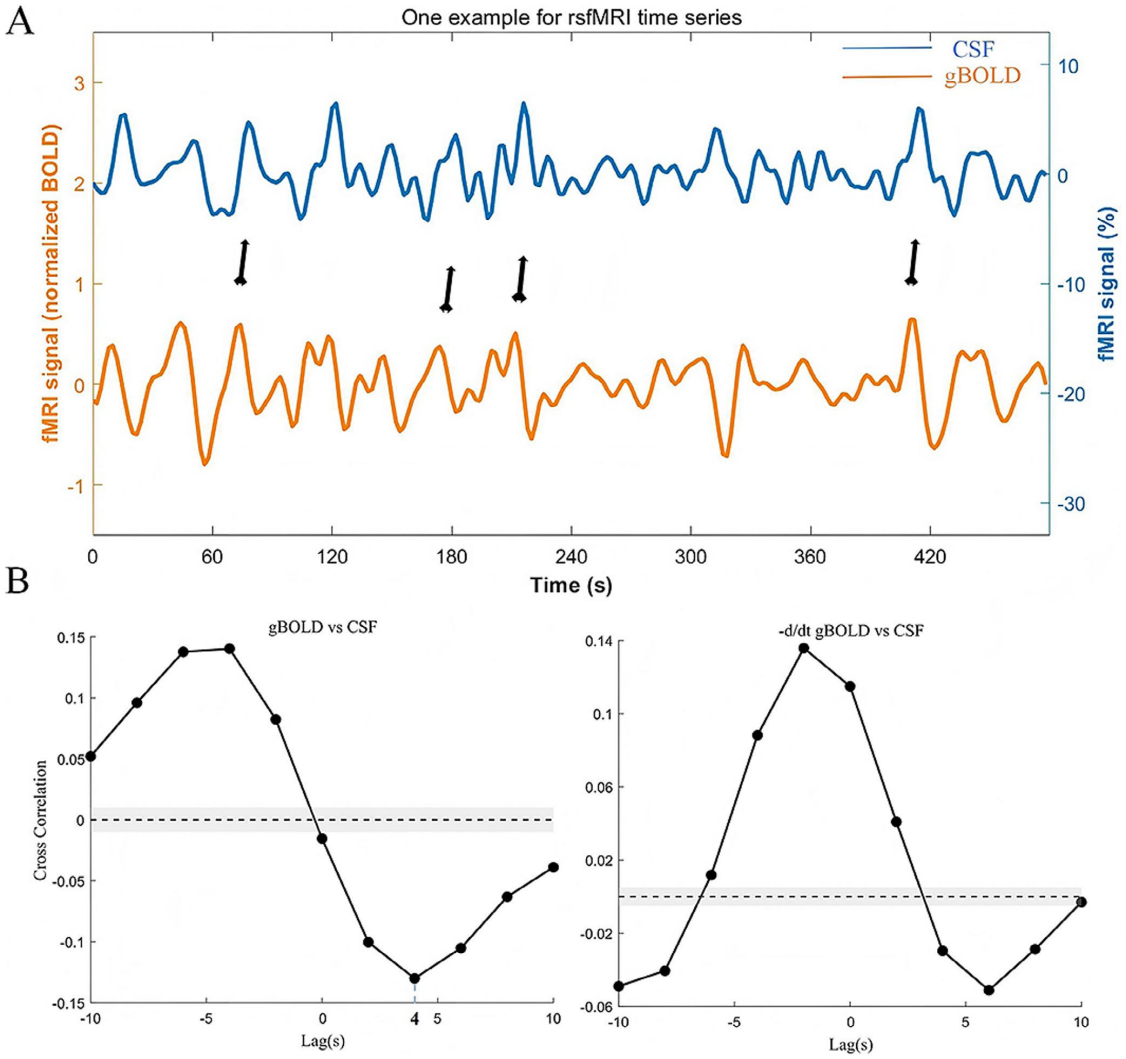
The systematic coupling between gBOLD and CSF signals measured by rs‐fMRI. (A) Changes in gBOLD and CSF signals were observed in a representative participant, showing that CSF signal peaks typically follow gBOLD signal peaks. (B) The averaged gBOLD‐CSF cross‐correlation function (*N* = 119) exhibited a positive peak at −4 s (*r* = 0.140, *p* < 0.001, permutation test with *n* = 10,000) and a negative peak at +4 s (*r* = −0.130, *p* < 0.001, permutation test with *n* = 10,000). The cross‐correlation function between the CSF signal and the negative derivative of the gBOLD signal showed a positive peak at −2 s (d/dt *r* = 0.136, *p* < 0.001, permutation test with *n* = 10,000). The black dashed line and gray shaded area indicate the 95% confidence interval of the mean correlation derived from shuffled signals.

We observed a significant cross‐correlation between the gBOLD and CSF signals in all participants. This correlation showed a clear temporal pattern, with the CSF signal peak typically following the gBOLD signal peak. The average cross‐correlation exhibited a positive peak at a −4 s lag (*r* = 0.140, *p* < 0.001) and a negative peak at a +4 s lag (*r* = −0.130, *p* < 0.001). Similar results were also observed across different windows (Figure S1). Furthermore, the cross‐correlation function between the negative derivative of the gBOLD signal and the CSF signal exhibited a significant positive peak at a −2 s lag (d/dt *r* = 0.136, *p* < 0.001) (Figure 2). These results indicate a systematic coupling between gBOLD fluctuations and CSF dynamics, consistent with previously reported findings. The two radiologists showed excellent agreement in the gBOLD‐CSF coupling measurements, with an ICC of 0.881.

### 3.3 Comparisons of Glymphatic‐Related Imaging Indices

**FIGURE 3:**
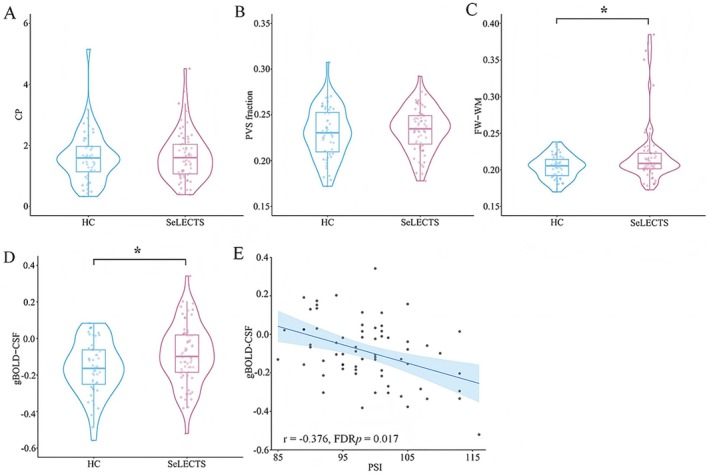
Alterations in glymphatic‐related MRI metrics in SeLECTS and correlations with cognitive function. Group differences in CP volume (A), PVS fraction (B), FW‐WM (C), and gBOLD‐CSF coupling strength (D) were compared between the HC and SeLECTS groups. The SeLECTS group showed a significant increase in FW‐WM and a significant decrease in gBOLD‐CSF coupling strength (less negative). (E) gBOLD‐CSF coupling was significantly negatively correlated with PSI. *Significant group differences, *p* < 0.05. CP, choroid plexus; CSF, cerebrospinal fluid; FW‐WM, white matter free water; PSI, processing speed; PVS, perivascular spaces.

Group comparisons revealed that the SeLECTS group had significantly higher FW‐WM values (FDR*p* = 0.042) but lower gBOLD‐CSF coupling strength (FDR*p* = 0.042) than the HC group. However, no significant between‐group differences were found in CP volume (FDR*p* = 0.782) or PVS fraction (FDR*p* = 0.211). Additionally, a significant negative correlation was observed between gBOLD‐CSF coupling and PSI (*r* = −0.376, FDR*p* = 0.017) (Figure 3).

### 3.4 Differences in TBSS and Functional Network Properties Between Groups

After adjusting for age, sex, and education, no significant differences in FAt and MDt were observed between the two groups.

**FIGURE 4:**
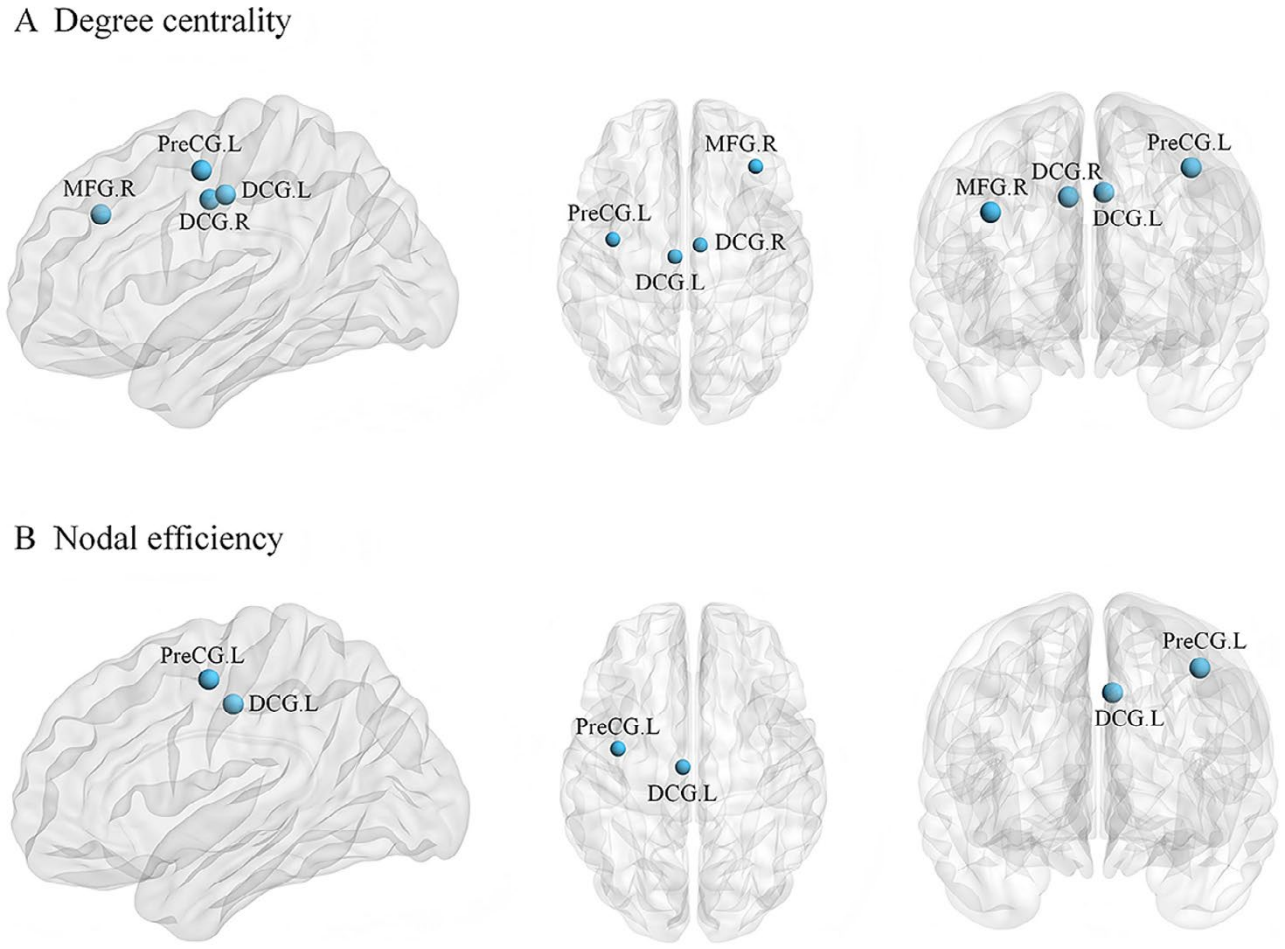
Brain regions showing altered nodal properties in functional brain networks. The blue color indicates decreased nodal properties in the SeLECTS group. DCG, median cingulate and paracingulate gyri; L, left; MFG, middle frontal gyrus; PreCG, precentral gyrus; R, right.

**FIGURE 5:**
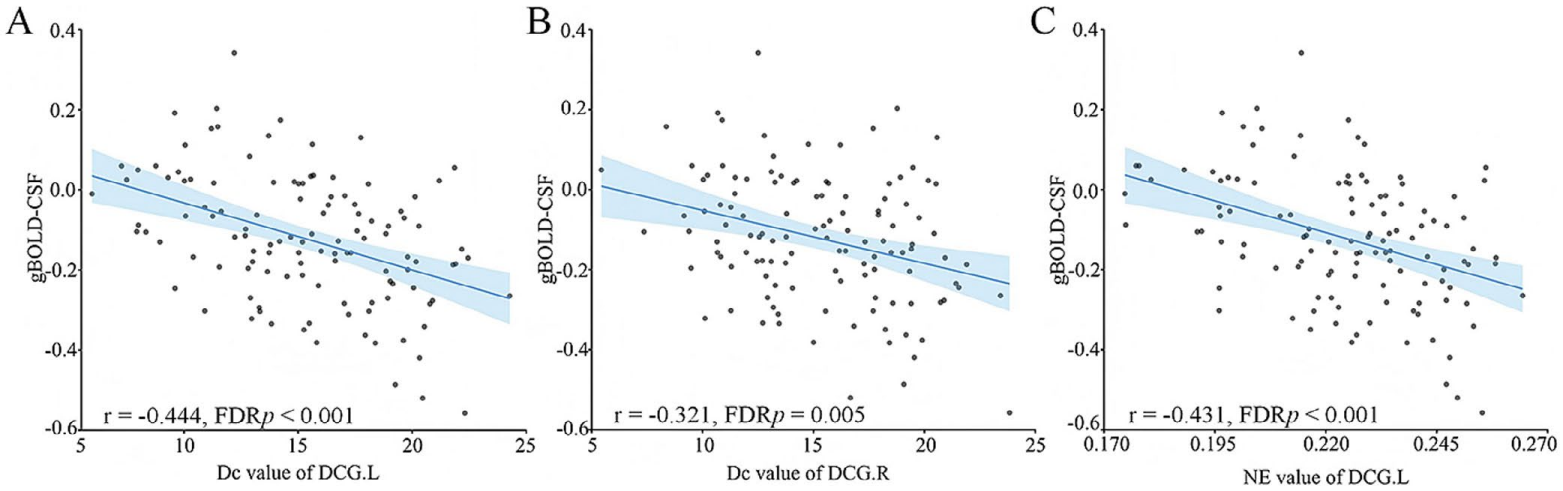
Associations between gBOLD‐CSF coupling and network properties. (A‐C) gBOLD‐CSF coupling was significantly negatively correlated with the Dc values of the bilateral DCG and the NE value of the left DCG. Dc, degree centrality; DCG, left median cingulate and paracingulate gyri; NE, nodal efficiency.

Graph theoretical analysis revealed that both the SeLECTS and HC groups exhibited small‐world network properties (*γ* > 1, *λ* ≈1, *σ* > 1). However, compared with the HC group, the SeLECTS group demonstrated significantly lower Cp (*p* = 0.006) and Lp (*p* = 0.043) values, alongside a higher Eg value (*p* = 0.022) (Table 2). At the nodal level, the SeLECTS group displayed significantly decreased Dc values in the left precentral gyrus (PreCG), right middle frontal gyrus (MFG), and bilateral median cingulate and paracingulate gyri (DCG), as well as decreased NE values in the left PreCG and left DCG, compared with the HC group (*p* < 0.05, Bonferroni‐corrected) (Figure 4). Validation analysis indicated that the alterations in network properties between the two groups were consistent across different sparsity ranges (Figures [Link], [Link], [Link], [Link]). The gBOLD‐CSF coupling was significantly negatively correlated with the Dc values of the left DCG (*r* = −0.444, FDR*p* < 0.001) and right DCG (r = −0.321, FDR*p* = 0.005), as well as the NE value of the left DCG (*r* = −0.431, FDR*p* < 0.001) (Figure 5).

**TABLE 2:** Comparison of AUC values for network properties between the HC and SeLECTS groups.

Network properties	HC (*n* = 52)	SeLECTS (*n* = 67)	Statistics	*p*
*σ*	0.552 ± 0.104	0.584 ± 0.104	−1.745	0.084
γ	0.612 ± 0.118	0.647 ± 0.118	−1.750	0.083
Cp	0.203 ± 0.007	0.199 ± 0.007	2.810	0.006*
λ	0.381 ± 0.006	0.381 ± 0.006	0.333	0.740
Lp	0.724 ± 0.035	0.709 ± 0.035	2.044	0.043*
Eg	0.181 ± 0.007	0.184 ± 0.007	−2.326	0.022*
Eloc	0.257 ± 0.006	0.257 ± 0.006	0.006	0.995

*Note:* Data are presented as mean ± SD.

Abbreviations: γ, normalized clustering coefficient; λ, normalized characteristic path length; *σ*, small‐worldness; Cp, clustering coefficient; Eg, global efficiency; Eloc, local efficiency; HC, healthy controls; Lp, characteristic path length; SeLECTS, self‐limited epilepsy with centrotemporal spikes.

* Significant group differences, *p* < 0.05.

### 3.5 Mediation Analysis

**FIGURE 6:**
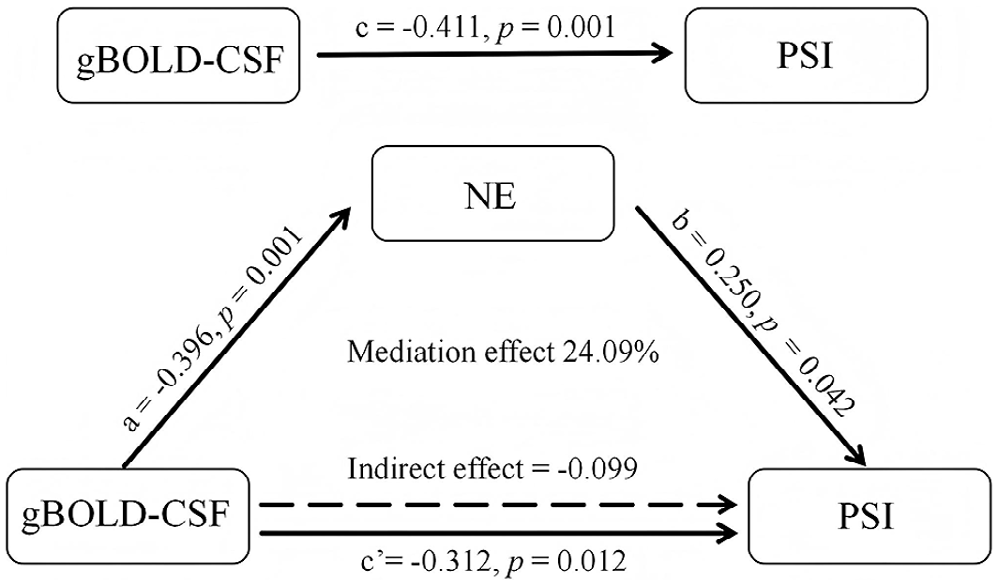
The mediation model showed that the NE value of the left DCG partially mediated the association between gBOLD‐CSF coupling and PSI, accounting for 24.09% of the total effect. NE, nodal efficiency; PSI, processing speed index.

The mediation analysis indicated that the NE value of the left DCG partially mediated the association between gBOLD‐CSF coupling and processing speed, accounting for 24.09% of the total effect (95% CI = −0.196 to −0.020) (Figure 6).

## 4 Discussion

This preliminary study aimed to systematically integrate multiple glymphatic‐related imaging metrics with functional network topological properties in drug‐naive children with SeLECTS. The main findings are as follows: (1) significant alterations in gBOLD‐CSF coupling and FW‐WM, (2) disrupted functional network topology at both the global and nodal levels, and (3) the mediating role of the reduced NE in the left DCG in the association between gBOLD‐CSF coupling and processing speed. These findings provide novel insights into the neuropathological basis of cognitive dysfunction in children with SeLECTS from the perspectives of cerebral waste clearance and brain network organization.

gBOLD signal fluctuations within the 0.01–0.1 Hz range are associated with slow vasomotor pulsations, which can drive CSF flow [27]. In the present study, the SeLECTS group exhibited significantly reduced gBOLD‐CSF coupling. This alteration may be attributed to several mechanisms. First, endothelial dysfunction, BBB disruption, and sustained hypertension following epileptic seizures may alter vascular compliance and weaken arterial pulsatility, thereby disrupting the highly coordinated process between vascular and fluid oscillations [28, 29]. Second, abnormal epileptic discharges in SeLECTS may induce asynchronous vasomotor responses in local cerebral vessels, leading to impaired neurovascular coupling [30]. Third, given that glymphatic waste clearance is most active during sleep, sleep disturbances commonly observed in children with epilepsy may further impair this process [31, 32].

Our findings are in line with those of recent studies. A study on temporal lobe epilepsy similarly revealed that the cross‐correlation function between gBOLD and CSF signals exhibited a maximum negative peak at a + 4 s lag. Compared with the HC group, patients with temporal lobe epilepsy exhibited significantly reduced gBOLD‐CSF coupling strength, further suggesting a seizure‐induced impairment of neurofluid dynamics [33]. In addition, from a structural perspective, Yin et al. reported a reduced DTI‐ALPS index in patients with SeLECTS, reflecting altered water diffusivity along perivascular pathways and providing indirect support for disturbances in CSF‐ISF exchange [34]. Importantly, our study also showed that the correlation between gBOLD‐CSF coupling and processing speed remained significant after FDR correction, highlighting that gBOLD‐CSF coupling may serve as a promising neuroimaging marker linking metabolic clearance processes to core cognitive deficits in SeLECTS. Nevertheless, additional clinical evidence and experimental validation are required to strengthen these conclusions, particularly in pediatric populations.

Additionally, a significant increase in FW‐WM was observed in the SeLECTS group. This observation may be partially attributed to the underlying pathophysiological mechanisms of SeLECTS. Specifically, seizure‐induced BBB disruption leads to the leakage of albumin and inflammatory mediators into the interstitium, thereby increasing the extracellular water content and impairing fluid circulation [35]. Moreover, recurrent seizures and neuroinflammation cause reactive astrogliosis and disrupt AQP4 polarization, which compromises CSF‐ISF exchange [36]. This disruption leads to the accumulation of metabolic waste, including excessive glutamate, phosphorylated tau protein, and pro‐inflammatory cytokines, ultimately disturbing ion homeostasis and impairing neuronal function. Collectively, these pathological changes disrupt the balance between excitation and inhibition, thereby increasing seizure susceptibility and establishing a vicious cycle [37, 38, 39, 40]. However, no significant between‐group differences were observed in FAt or MDt. The increased FW may therefore reflect alterations in extracellular water content without accompanying detectable white matter microstructural changes, which contrasts with previous DTI findings [41, 42]. This discrepancy may be explained by the fact that conventional diffusion metrics can be confounded by increased extracellular water content. Conversely, the bitensor model used in this study eliminated the FW component, thereby providing a more sensitive assessment of microstructural changes in white matter. Furthermore, no significant alterations in CP volume or PVS fraction were observed. Both measures are frequently altered in neurodegenerative and neuroinflammatory diseases, such as multiple sclerosis, Parkinson's disease, and Alzheimer's disease, and may reflect disease severity and systemic inflammation [43, 44, 45, 46, 47]. Unlike previous studies, the present study focused on patients with early‐stage, drug‐naive SeLECTS. These findings suggest that the observed alterations in imaging metrics are more likely to reflect functional rather than structural disturbances in CSF flow.

The human brain is organized as a complex network with small‐world properties, enabling efficient information transfer through the maintenance of an optimal balance between functional integration and segregation [48]. In the present study, although both groups exhibited a small‐world topology, the SeLECTS group demonstrated significantly lower Cp and Lp values and a higher Eg value compared with the HC group, suggesting a more random network topology. Specifically, a reduction in Cp reflects a reduced capacity for local information processing, which is critical for complex cognitive functions. Conversely, decreased Lp and increased Eg indicate enhanced global efficiency of information transfer, which contrasts with the findings of several studies [49, 50]. This discrepancy may reflect a compensatory mechanism in the early stage of the disease. It may also be influenced by patient heterogeneity in terms of disease duration and medication status, as well as differences in network construction approaches, given that previous studies have primarily used cortical morphometric features or DTI‐based structural networks. Additionally, compared with the HC group, the SeLECTS group exhibited significantly lower Dc and NE values across multiple brain regions, reflecting decreases in nodal importance and communication efficiency, respectively. These reductions were found in the PreCG, a key region within the sensorimotor network, suggesting impaired integration of motor‐related information. This impairment may be closely related to clinical motor symptoms, such as facial and oropharyngeal muscle twitching [51]. Moreover, the affected regions included the MFG, which plays critical roles in executive functions such as attentional regulation and working memory, and the DCG, which is involved in emotional regulation and cognitive control processes. Collectively, these findings indicate widespread alterations in functional network topology, which may provide a neuropathological basis for the clinical deficits observed in patients with SeLECTS [52, 53].

Another important finding was that the NE value of the left DCG mediated the association between gBOLD‐CSF coupling and processing speed. Similar results were reported by Gao et al., who demonstrated that glymphatic dysfunction may influence cognitive performance through alterations in brain network efficiency [54]. These findings underscore that the coordination between effective waste clearance and efficient functional brain networks is vital for maintaining normal cognitive function. However, given the cross‐sectional design of the present study, these findings should be interpreted as statistical associations rather than definitive evidence of a causal pathway. Therefore, future longitudinal studies should be conducted to clarify these relationships.

This study has several limitations. First, the cross‐sectional design precludes causal inference, and the relatively small sample size may have limited the statistical power of the correlation and mediation analyses. Therefore, larger multicenter longitudinal studies are needed to further validate the present findings. Second, this study employed indirect neuroimaging markers to assess glymphatic‐related function and lacked validation with a gold standard. Therefore, the results should be interpreted with caution, and future studies should employ multimodal imaging or experimental validation to strengthen the mechanistic interpretation. Third, although a standardized MRI acquisition protocol was implemented, concurrent EEG monitoring to objectively assess vigilance states was not performed due to practical clinical challenges, thereby introducing a potential state‐related confounding factor. Future studies should integrate simultaneous EEG‐fMRI or other physiological monitoring approaches to control for the influence of vigilance states on gBOLD‐CSF coupling. Fourth, detailed electrophysiological and clinical variables reflecting disease heterogeneity, such as spike burden and EEG characteristics, were not systematically evaluated due to resource constraints. Future investigations incorporating these comprehensive clinical variables are warranted to strengthen the disease specificity of our findings. Finally, simultaneous sleep monitoring was not conducted. Future research incorporating comprehensive sleep assessments will help elucidate the complex interactions between sleep architecture and glymphatic function in patients with SeLECTS.

In summary, this study employed multiparametric MRI using complementary imaging markers to demonstrate that drug‐naive SeLECTS patients exhibit altered CSF flow dynamics, which are closely associated with brain network reorganization and cognitive dysfunction. Future studies should explore intervention strategies targeting the clearance of metabolic waste and the maintenance of microenvironmental homeostasis as potential approaches for improving disease prognosis.

## Funding

This study was supported by the Key Basic Research Program of Guizhou Province (Qiankehejichu‐ZK[2022]zhongdian 051), Talent Program for Future Famous Clinical Doctors of Zunyi Medical University (rc220211205), and Innovation Talent team of Science and Technology in Zunyi City (Zunshikerencai[2024]5).

## Disclosure

The authors have nothing to report.

## Ethics Statement

The study was approved by the ethics committee of Chengdu Women's and Children's Central Hospital review board ([2024]50).

## Consent

Written informed consent was obtained from all participants.

## Conflicts of Interest

The authors declare no conflicts of interest.

## Supporting information


**Figure S1:** Cross‐correlation function analysis of gBOLD‐CSF coupling across different window ranges revealed that the maximum negative peaks consistently occurred at a lag of +4 s.

**Figure S2:** Global properties were compared between the SeLECTS and HC groups across a sparsity range of 0.05–0.35 with a step size of 0.01.

**Figure S3:** Nodal properties were compared between the SeLECTS and HC groups across a sparsity range of 0.05–0.35 with a step size of 0.01. The blue color indicates decreased nodal properties in the SeLECTS group.

**Figure S4:** Global properties were compared between the SeLECTS and HC groups across a sparsity range of 0.10–0.40 with a step size of 0.01.

**Figure S5:** Nodal properties were compared between the SeLECTS and HC groups across a sparsity range of 0.10–0.40 with a step size of 0.01. The blue color indicates decreased nodal properties in the SeLECTS group.

**Table S1:** Correlation analysis of clinical variables with gBOLD‐CSF coupling and FW‐WM.
**Table S2:** Correlation analysis of significantly altered network properties with gBOLD‐CSF coupling and FW‐WM.

## Data Availability

The data that support the findings of this study are available from the corresponding author upon reasonable request.
