# Supplementary material for: Altered Glymphatic‐Related MRI Metrics and Brain Network Reorganization in Drug‐Naive Children With Self‐Limited Epilepsy With Centrotemporal Spikes
Source: CNS Neurosci Ther. 2026 Aug 12;32(8):e71079. doi: 10.1002/cns.71079 (PMC13469168; doi:10.1002/cns.71079)
Supplement: Supplementary file 1 [file CNS-32-e71079-s006.docx]

**Table S1.** Correlation analysis of clinical variables with gBOLD-CSF coupling and FW-WM

| Characteristics | gBOLD-CSF | | | FW-WM | | |
| --- | --- | --- | --- | --- | --- | --- |
|  | r | *p* | FDR*p* | r | *p* | FDR*p* |
| Disease duration | 0.064 | 0.613 | 0.785 | 0.013 | 0.917 | 0.978 |
| Total number of seizures | 0.092 | 0.468 | 0.749 | -0.076 | 0.553 | 0.785 |
| VCI | -0.205 | 0.104 | 0.349 | 0.118 | 0.352 | 0.668 |
| PRI | -0.067 | 0.597 | 0.785 | 0.072 | 0.572 | 0.785 |
| WMI | 0.170 | 0.180 | 0.524 | 0.033 | 0.796 | 0.895 |
| PSI | -0.376 | 0.002 | 0.017^*^ | 0.076 | 0.548 | 0.785 |
| FSIQ | 0.097 | 0.443 | 0.747 | -0.320 | 0.010 | 0.063 |
| Abbreviations: FSIQ, full-scale intelligence quotient; PRI, perceptual reasoning index; PSI, processing speed index; VCI, verbal comprehension index; WMI, working memory index. *Significant group differences, *p* < 0.05. | | | | | | |

**Table S2.** Correlation analysis of significantly altered network properties with gBOLD-CSF coupling and FW-WM

| Network properties | gBOLD-CSF | | | FW-WM | | |
| --- | --- | --- | --- | --- | --- | --- |
|  | r | *p* | FDR*p* | r | *p* | FDR*p* |
| **Global properties** |  |  |  |  |  |  |
| Cp | -0.231 | 0.013 | 0.067 | 0.041 | 0.659 | 0.811 |
| Lp | -0.108 | 0.247 | 0.658 | 0.001 | 0.994 | 0.994 |
| Eg | 0.150 | 0.109 | 0.349 | 0.005 | 0.958 | 0.989 |
| **Nodal properties** |  |  |  |  |  |  |
| **Degree centrality** |  |  |  |  |  |  |
| Left PreCG | -0.174 | 0.062 | 0.247 | 0.024 | 0.795 | 0.895 |
| Right MFG | -0.081 | 0.387 | 0.688 | -0.102 | 0.275 | 0.668 |
| Left DCG | -0.444 | 0.000 | <0.001^*^ | -0.092 | 0.323 | 0.668 |
| Right DCG | -0.321 | 0.000 | 0.005^*^ | -0.087 | 0.355 | 0.668 |
| **Nodal efficiency** |  |  |  |  |  |  |
| left PreCG | -0.177 | 0.058 | 0.247 | 0.022 | 0.811 | 0.895 |
| Left DCG | -0.431 | 0.000 | <0.001^*^ | -0.095 | 0.311 | 0.668 |
| Abbreviations: Cp, clustering coefficient; DCG, median cingulate and paracingulate gyri; Eg, global efficiency; Lp, characteristic path length; MFG, middle frontal gyrus; PreCG, precentral gyrus. *Significant group differences, *p* < 0.05. | | | | | | |

**Figure legends**

**Figure S1.** Cross-correlation function analysis of gBOLD-CSF coupling across different window ranges revealed that the maximum negative peaks consistently occurred at a lag of +4 s.

**Figure S2.** Global properties were compared between the SeLECTS and HC groups across a sparsity range of 0.05-0.35 with a step size of 0.01.

**Figure S3.** Nodal properties were compared between the SeLECTS and HC groups across a sparsity range of 0.05-0.35 with a step size of 0.01. The blue color indicates decreased nodal properties in the SeLECTS group.

**Figure S4.** Global properties were compared between the SeLECTS and HC groups across a sparsity range of 0.10-0.40 with a step size of 0.01.

**Figure S5.** Nodal properties were compared between the SeLECTS and HC groups across a sparsity range of 0.10-0.40 with a step size of 0.01. The blue color indicates decreased nodal properties in the SeLECTS group.
